# Bringing the treatable traits approach to primary care asthma management

**DOI:** 10.3389/falgy.2023.1240375

**Published:** 2023-09-20

**Authors:** Paul E. Pfeffer, Hitasha Rupani, Anna De Simoni

**Affiliations:** ^1^ Department of Respiratory Medicine, Barts Health NHS Trust, London, United Kingdom; ^2^ William Harvey Research Institute, Queen Mary University of London, London, United Kingdom; ^3^ Department of Respiratory Medicine, University Hospital Southampton NHS Foundation Trust, Southampton, United Kingdom; ^4^ Wolfson Institute of Population Health and Asthma UK Centre for Applied Research, Queen Mary University of London, London, United Kingdom

**Keywords:** asthma, personalised medicine, inducible laryngeal obstruction, biomarker, adherence, breathing pattern disorder

## Abstract

Asthma continues to be a major cause of illness with a significant mortality, despite its increasing range of treatments. Adoption of a treatable traits approach in specialist centres has led to improvements in control of asthma and reduced exacerbations in patients with severe asthma. However, most patients with this illness, particularly those with mild-to-moderate asthma, are cared for in primary care according to guidelines that emphasise the use of pharmacotherapeutic ladders uniformly implemented across all patients. These pharmacotherapeutic ladders are more consistent with a “one-size-fits-all” approach than the treatable traits approach. This can be harmful, especially in patients whose symptoms and airway inflammation are discordant, and extra-pulmonary treatable traits are often overlooked. Primary care has extensive experience in patient-centred holistic care, and many aspects of the treatable traits approach could be rapidly implemented in primary care. Blood eosinophil counts, as a biomarker of the treatable trait of eosinophilia, are already included in routine haematology tests and could be used in primary care to guide titration of inhaled corticosteroids. Similarly, poor inhaler adherence could be further assessed and managed in primary care. However, further research is needed to guide how some treatable traits could feasibly be assessed and/or managed in primary care, for example, how to best manage patients in primary care, who are likely suffering from breathing pattern disorders and extra-pulmonary treatable traits, with frequent use of their reliever inhaler in the absence of raised T2 biomarkers. Implementation of the treatable traits approach across the disease severity spectrum will improve the quality of life of patients with asthma but will take time and research to embed across care settings.

## Introduction

**Figure 1:**
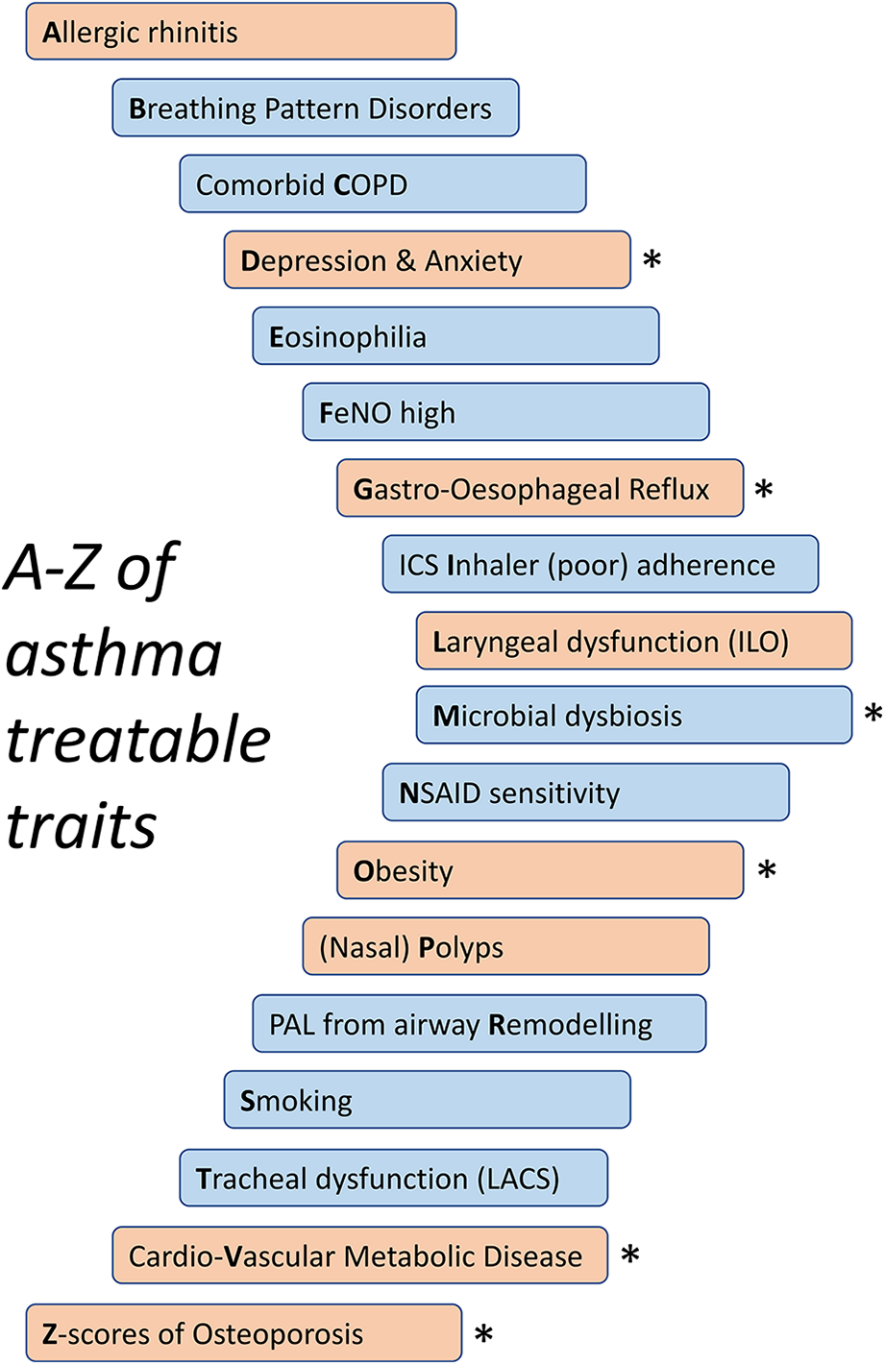
An abbreviated A–Z of treatable traits in asthma. Pulmonary traits in blue and extra-pulmonary traits in orange. LACS, large-airway collapse with symptoms; PAL, persistent airflow limitation. *Potential corticosteroid exposure-associated traits.

The concept that individual clinical characteristics, “traits,” are the key determinant of appropriate patient management, with treatment options dependent on the traits identified (Figure 1), is a prevailing paradigm of care in severe asthma management worldwide. Assessment of trait biomarkers has been necessary for selecting patients likely to respond to most monoclonal biologics. Furthermore, the treatable traits approach with systematic patient assessment undertaken at severe asthma centres significantly improves asthma outcomes even in patients not started on biologics, with assessment and treatment for traits such as non-adherence and breathing pattern disorders (BPD) resulting in improved clinical outcomes (1, 2).

This model of personalised medicine, in contrast to a one-size-fits-all model, has taken 20 years to become established as a standard of care in severe asthma, and the vast majority of relevant research and implementation experience in treatable traits has been in severe asthma. However, most patients with asthma are diagnosed and managed in primary care where treatment is primarily according to guidelines such as the GINA and BTS/SIGN pharmacotherapeutic ladders (3, 4). Whilst the pharmacotherapeutic ladders are a pragmatic necessity and are successful in controlling asthma in the majority of patients (5), they have limitations. For example, such a one-size-fits-all approach can lead to breathlessness caused by comorbid conditions such as BPD mistakenly being treated pharmacologically with escalation of asthma medications. A growing body of new research evidence and experience challenges whether one-size-fits-all care is totally effective in treating mild-to-moderate asthma. In addition, many patients with unrecognised, uncontrolled severe asthma are managed in primary care (6). Improvements in mortality and morbidity of asthma at a population level have stagnated, and it may be that translating the treatable traits approach to primary care could lead to population-level improvements in asthma management (7).

Based on a recent study of over 30,000 patients with asthma from North-East London, over 6,000 patients with mild asthma (low exacerbation frequency, low asthma medication step, low probability of being eosinophilic) were prescribed six or more short-acting beta-agonist (SABA) reliever inhalers in the preceding year, suggesting high levels of symptoms in the absence of severe disease (8). Management approaches other than the pharmacotherapeutic ladders would likely be more appropriate to treat such patients that are commonly encountered in primary care.

In this article, we discuss the treatable traits approach and raise the question of how this personalised medicine approach may be implemented in primary care. We first review the benefits of using the treatable traits approach in the management of severe asthma and then discuss how the one-size-fits-all approach leads to inappropriate and potentially harmful management of patients with mild-to-moderate asthma, before considering specific treatable trait diagnoses and interventions and how they could be delivered in primary care (Table 1), the setting wherein most patients with asthma are managed. We aim to highlight the current gaps in primary care management of asthma and the research questions that need to be addressed to further facilitate implementation of the treatable traits approach in primary care (Table 1).

**Table 1:** Major treatable traits in asthma, considerations for implementation in primary care, and related research questions.

Treatable trait	Considerations for implementation in primary care	Research questions and considerations
Pulmonary treatable traits		
Eosinophilia (T2-high)	• Blood eosinophil counts available in primary care in most regions to guide increases in inhaled corticosteroids	
High FeNO (T2-high)	• Emerging biomarker for identifying patients for ICS adherence review and guiding increases in inhaled corticosteroids; limited but increasing availability in primary care	
Persistent airflow limitation	• Variable availability of spirometry in primary care in some regions but important for guiding prescription of inhaled LAMA	• Pragmatic criteria for identifying asthma patients in primary care who would benefit from the addition of LAMA inhalers
Poor adherence to preventer inhalers	• Medication possession ratio easy to assess in primary care	• How to best implement inhaler adherence interventions in primary care
Chronic breathlessness symptoms	• Potential role of pulmonary rehabilitation in patients with asthma	• Optimal components and delivery methods for pulmonary rehabilitation in patients with asthma
Psychological dependence on SABA inhalers		• How to best manage patients with high SABA use despite no evidence of airway inflammation or airflow obstruction
BPD	• Nijmegen score will detect patients with hyperventilation syndrome though this is only one of several types of BPD	• Diagnostic tools for BPD that are implementable in primary care • Resource-efficient management strategies for BPD in primary care
Extra-pulmonary treatable traits		
ILO	• Pittsburgh VCD Index could potentially be used in primary care as a screening tool for patients with possible ILO	• Diagnostic tools for ILO feasible for use in primary care • Resource-efficient management strategies for ILO in primary care
Allergy and allergic rhinitis	• All routinely managed in primary care but potentially overlooked during asthma reviews	• Whether inclusion of extra-pulmonary treatable traits in templates for primary care asthma management improves the diagnosis and management of these comorbidities
Depression and anxiety		
Gastro-oesophageal reflux		
Obesity		
Cardiovascular/metabolic disease		
Osteoporosis		

BPD, breathing pattern disorder; ILO, inducible laryngeal obstruction; LAMA, inhaled long-acting muscarinic antagonist; ICS, inhaled corticosteroids.

It is important to acknowledge that primary care has extensive experience in providing holistic care of similar manner to that advocated by the treatable traits approach, but applied to general health rather than specifically to asthma, with strong relationship-based care (9–11). In this article, research questions are raised regarding how some pulmonary treatable traits could best be assessed and managed in primary care. However, primary care already has the experience in managing most extra-pulmonary treatable traits, even though awareness of their importance as an integral part of asthma management potentially needs to be raised.

## Implementation of treatable traits approach in severe asthma

### Treatable traits approach based on T2 inflammation

Airway inflammation is the hallmark of asthma and underlies the pathophysiological changes seen within the asthmatic airways. It is frequently categorised into type 2 (T2)-high asthma or T2-low asthma based on the predominance of cytokines and inflammatory cells within the airways. T2-high asthma is characterised by eosinophilic inflammation, and recent analyses indicate that it is the predominant phenotype in patients with mild-to-moderate (12) and severe (13) asthma. After a clinical trial of anti-IL-5, anti-eosinophil, biologic therapy in a broad population of asthmatic patients reported a non-significant clinical effect (14), Pavord and colleagues established that anti-IL-5 therapy would yield clinically significant responses when given to patients with severe asthma *characterised by the treatable trait of eosinophilia*, establishing the importance of management guided by the treatable traits in asthma (15, 16).

Eosinophilia is now well established as a biomarker of patients likely to respond to anti-IL-5 biologic therapies. It is also closely linked to risk of exacerbation and asthma control, with higher levels of blood eosinophils associated with an increased risk of exacerbation and worse asthma control (17). Similarly, raised fractional exhaled nitric oxide (FeNO) is recognised as a biomarker of IL-4/IL-13-mediated T2 airway inflammation, a treatable trait associated with response to dupilumab (18). However, more importantly, FeNO is also increasingly recognised as a biomarker of both (i) responsiveness to inhaled steroid therapy and (ii) the treatable trait of poor adherence to inhaled corticosteroids (ICS) in patients with asthma, indicating those who may benefit from a personalised approach to improve treatment adherence, such as the period of digital inhaler monitoring (19).

The blood eosinophil count and FeNO are established as biomarkers of airway inflammation in asthma with increased levels of these biomarkers associated with an increased risk of exacerbation and a decline in lung function (17, 20). Blood eosinophil counts, as part of the white blood cell count differential on routine haematology blood counts, can be checked in primary care in most international regions, even though they may not have been checked within a recent time-frame in many patients even in regions where local laboratories routinely report eosinophil counts (12). Knowledge and availability of FeNO in both primary and secondary care are variable across different healthcare systems and limited in some countries (21). However, as the evidence base for FeNO grows, its use in primary care is increasing (22), and it is implementable in primary care (23).

### Treatable traits approach beyond T2 inflammation

Importantly, the treatable traits approach utilised in severe asthma clinics extends beyond assessment and targeted treatments of T2 inflammation (24). Systematic assessment of patients with severe asthma includes assessment for and management of an A to Z of treatable traits: from allergy through eosinophilia and obesity to (Z-scores of) osteoporosis (Figure 1). These treatable traits include those that relate to the lungs and airways (pulmonary traits) and those that extend beyond the lungs (extra-pulmonary traits).

This approach in UK Severe Asthma Centres is associated with a reduction in exacerbations and maintenance oral steroid use, as well as symptomatic improvement (1). Although the improvement is higher in those commenced on biologic therapies (not available in primary care), a major improvement in patients whose treatable traits-guided management does not include biologic therapy (for example, a 75% reduction in exacerbation in the former, and 54% in the latter) is still noted (1).

However, this approach requires a multidisciplinary team in each severe asthma clinic including psychologists, physiotherapists, speech (laryngeal) therapists, pharmacists, dieticians, as well as specialist doctors and nurses (25). Whilst these professionals do work within the community, there is insufficient resource within primary care to expect all, or even most, patients with asthma to be assessed by each of them. Nevertheless, when the “treatable traits” approach was first coined, it was not intended to be reserved for severe asthma centres, with the authors concluding that such a strategy should be feasible and beneficial in primary care (26).

## Harms associated with non-implementation of the treatable traits approach in primary care

Management of mild-to-moderate asthma in primary care is largely dictated by therapeutic “ladders” in which patient pharmacotherapy, after the assessment of inhaler technique and self-management knowledge, is escalated up the ladder in response to uncontrolled symptoms or exacerbations and de-escalated in response to periods of stability. These pharmacotherapy ladders typically start with low-dose ICS, and as the ladder is ascended, long-acting beta-agonist bronchodilators (LABA) are added in the form of an ICS–LABA combination inhaler, followed by steadily increasing the strength of ICS in the ICS–LABA inhaler. In addition to these, trials of leukotriene receptor antagonists and separate inhaled long-acting muscarinic antagonist (LAMA) inhalers may be added. However, ultimately, when following a pharmacotherapeutic ladder guideline (3, 4), patients with uncontrolled symptoms or attacks of asthma-like symptoms will tend to ascend the ladder until they are on high-dose inhaled corticosteroids, regardless of whether their symptoms are caused by asthma or other comorbid conditions listed in Figure 1 (27–29).

This approach is predicated on the symptoms, whether day-to-day or episodic attacks, being preventable with inhaled corticosteroids and the magnitude of the symptoms being proportionate to the degree of steroid-responsive pathology in the airways. Symptoms proportionate and concordant to T2 airway inflammation are observed in many patients. However, major patient groups with symptoms discordant to airways inflammation, the target of inhaled corticosteroids, with either a high symptom burden in the absence of elevated T2 biomarkers or elevated T2 biomarkers and high exacerbation risk but low symptom burden outside of exacerbations, are reported (30). These patients with discordant symptoms and inflammation are at risk of harm from management based on implementation of the “one-size-fits-all” pharmacotherapeutic ladders.

### Risks in inflammation-predominant patients

One patient group with discordance is those with higher inflammation than symptoms, with significant airway inflammation, but not day-to-day symptoms. The relative absence of day-to-day symptoms can lead to treatment with inhaled corticosteroids of low strength, insufficient to control airway inflammation, and as a result, episodic severe exacerbations. Recurrent exacerbations negatively affect the quality of life of the patients, lead to absence in work and/or education and significant use of healthcare resources. Oral steroids, used to treat exacerbations, also have numerous side effects including increased risk of sepsis, osteoporosis, diabetes, and cardio-metabolic diseases (31, 32). Over 20 years ago, Green and colleagues reported biomarkers of airway inflammation to be better than symptoms at directing titration of inhaled corticosteroid strength to prevent exacerbations (33). Whilst their pioneering research used sputum eosinophil counts, more recent research with blood eosinophil counts and FeNO, surrogate T2 biomarkers of sputum eosinophilia, has confirmed the effectiveness of a biomarker-directed approach to titrating corticosteroids in asthma (34, 35). The treatable traits approach, separating out treatable traits of airway inflammation and of breathlessness, is therefore safer and protects the patients from exacerbations, by directing appropriately higher doses of inhaled corticosteroids where needed.

### Risks in symptom-predominant patients

The other patient group with discordance between symptoms and T2 airway inflammation are those with high levels of symptomatology without high levels of airway inflammation. Symptom-directed escalation of inhaled corticosteroid strength is ineffective at managing symptoms in these patients and leads to twin harms of (i) inappropriate high-strength ICS and (ii) failure to manage day-to-day symptoms.

Whilst high-strength ICS has a favourable benefit-to-risk ratio in patients who require high-strength inhaled corticosteroids for controlling airway inflammation and exacerbation risk, with far fewer adverse effects than oral steroids, potentially harmful side effects to higher-strength ICS, such as adrenal suppression (36) and susceptibility to certain airway infections, are found (37). As such, high-dose inhaled steroids in the absence of airway inflammation are likely to have a less favourable benefit-to-risk ratio.

The failure of inhaled corticosteroids to control asthma-like symptoms in some patients is of major importance in itself. In some patients, steroid-resistant airway inflammation due to oxidative stress, for example, from smoking (38), or due to airway microbial dysbiosis, which is defined as an imbalance in normal airway microbial populations (39), explains the discordance between symptoms and steroid-sensitive T2 airway inflammation. However, in large populations of patients with mild-to-moderate asthma, comorbid causes of breathlessness may be a more important explanation of discordance between asthma-like symptoms and T2 biomarkers of airway inflammation. Importantly, pathologies such as BPD, including hyperventilation, and laryngeal dysfunction, including inducible laryngeal obstruction (ILO), have similar symptoms to asthma and are often difficult to differentiate (27, 40).

Whilst asthma and asthma-like disorders such as BPD and ILO have at times been treated as mutually exclusive diagnoses, in reality many patients have both asthma and asthma-like comorbidities (41). As such, a confirmed diagnosis of asthma does not mean that the current symptoms of a patient are due to their asthma.

Failure to diagnose asthma-like comorbidities is likely a major contributor to a large number of patients overusing SABA inhalers for frequent breathlessness symptoms. In particular, based on our recent observational study, the majority of patients with asthma over-prescribed SABA inhalers were non-eosinophilic (8, 42). Whilst over-reliance on SABA is clearly dangerous in exacerbation-prone patients, even in patients who are not exacerbation prone, overuse of SABA is potentially harmful. SABA overuse is associated with poor asthma control (43) and extra-pulmonary side effects of excess *β*-adrenergic stimulation potentially including cardiac disease and increased risk of sepsis (44, 45).

In addition to being harmful, inappropriate use of asthma medications attempting to treat symptoms not due to asthma is unsuccessful leaving patients with a high symptom burden and at risk of side effects from unnecessary treatments. Untreated breathlessness is a major cause of psychological distress for affected patients and associated with significant functional limitation and morbidity (46).

### Harms resulting from not managing extra-pulmonary treatable traits

Asthma is associated with many other conditions, such as osteoporosis and obesity, and other conditions associated with systemic corticosteroid toxicity. These not only have their own harmful impacts on patients but can also worsen asthma outcomes. The impact of these comorbidities is modifiable, and therefore they are considered extra-pulmonary treatable traits in asthma. However, in primary care consultations that focus on inhaled pharmacotherapy, management of other comorbid conditions such as osteoporosis or gastro-oesophageal reflux can be overlooked in terms of being integral to asthma management—the complications of these comorbidities are highly prevalent at referral to severe asthma centres (47).

For example, untreated osteoporosis is associated with fragility fractures, with a significantly raised incidence rate in asthmatic patients (48). Osteoporotic vertebral fractures can negatively affect respiratory mechanics and lung function (49), which is of significant impact in patients with impaired lung function due to asthma. Similarly, obesity itself can additionally impair lung function (50) and further promote lung inflammation. Nasal inflammation is common in asthma and, if untreated, is associated with lower airway inflammation, in keeping with a unified upper and lower airway, and impaired nasal breathing, worsening asthmatic breathlessness (51).

## Implementation of the treatable traits approach in primary care

### Treatable traits approach to inhalers in primary care

The advent of triple combination inhalers (ICS–LABA–LAMA) containing either medium-strength or high-strength inhaled corticosteroids potentially provides a timely moment to introduce a treatable traits approach to inhaled therapy to primary care. For a patient with uncontrolled asthma on a medium-strength ICS–LABA inhaler, there is no longer a single option for the next step up the treatment ladder, but a branching ladder with options to step up either to a high-strength ICS–LABA inhaler or to a medium-strength ICS–LABA–LAMA combination inhaler.

Evidence has shown that patients with persisting blood eosinophilia are most likely to favourably respond to a step-up to high-strength ICS–LABA (52). Blood eosinophil counts are not only feasible in primary care in most regions but are also often checked with results available. For example, based on our recent evaluation of patients over-prescribed SABA inhalers, over 75% had a blood count with eosinophil count measured in the last 2 years (8). Guidance whether to consider stepping up from a medium-strength ICS–LABA to a high-strength ICS–LABA based on blood eosinophil count could be implemented in primary care.

The other biomarker of T2-mediated airway inflammation, FeNO, also increasingly shows a potential for use in primary care (23), such as to aid titration of inhaled corticosteroid strength. FeNO testing is easy to conduct and interpret, with the potential for use in primary care to be cost-effective whilst improving asthma control (53).

For stepping up from medium-strength ICS–LABA to medium-strength ICS–LABA–LAMA, evidence suggests a few criteria based on persistent abnormal lung function, e.g., airflow limitation on spirometry (54, 55). However, in some global regions, current capacity constraints for spirometry exist, and in these regions, the feasibility of repeat spirometry in primary care to guide stepping up to LAMA therapy is questionable. *Research on pragmatic criteria for identifying patients in primary care who would benefit from an addition of a LAMA inhaler is now needed*.

However, before the implementation of any escalation or step-up of treatment, it is necessary to review the potential treatable traits of poor inhaler technique and/or poor adherence to the current prescribed preventer treatment. Adherence can be easily evaluated in primary care by reviewing the number of prescriptions for ICS-containing inhalers collected by the patient over a 6–12-month period and comparing it to the number of inhalers that should have been collected (known as the medication possession ratio, MPR) (56). Different interventions can then be delivered to address different causes for poor adherence. For example, patients on twice-daily inhalers who forget to take doses at one particular time of the day may benefit from switching to a once-daily preventer inhaler. Patients whose health beliefs are in keeping with only using inhalers when symptomatic will likely benefit from switching to maintenance and reliever therapy (MART), provided that their airway inflammation and symptoms are in proportion. Both of these inhaler regimens are available in primary care. Reviewing inhaler devices and reducing inhaler device polypharmacy can also help improve adherence and inhaler technique. Digital inhalers that link to a patient's smartphone are becoming available and through reminders help patients reduce accidental non-adherence. *Implementation research on how to best implement inhaler adherence interventions in primary care is now needed*.

### Diagnosis of breathing pattern disorders and laryngeal dysfunction in primary care

BPD are a common cause of breathlessness in both patients with asthma and those without (41). Importantly, hyperventilation is only one type of BPD, with other types such as apical-predominant breathing, which is of major clinical importance (57). The gold standard for diagnosing BPD is assessment by a specially trained respiratory physiotherapist using Manual Assessment of Respiratory Motion (MARM) (58) and, for the measure of objective indices, assessment by Cardio-Pulmonary Exercise Test (CPET) (59). However, neither of these are feasible for routine patient assessment in primary care. Whilst the Nijmegen Questionnaire has reasonable sensitivity and specificity for diagnosing hyperventilation syndrome, it has poor utility for diagnosing other forms of BPD. However, it is still useful to highlight the presence of hyperventilation, helping avoid unnecessary pharmacotherapeutic escalation.

The Breathing Pattern Assessment Tool (BPAT) has high sensitivity and specificity for diagnosing BPD in asthma and other diseases and is quick to conduct (60, 61). However, based on publications to date, its use has only been evaluated when conducted by respiratory specialists. Whether the diagnosis of BPD using the BPAT assessment could be done by staff in primary care, after a short period of training, with similarly good sensitivity and specificity is uncertain, and *research regarding the feasible assessment measures for BPD in primary care is needed*.

ILO, where the larynx pathologically closes on inspiration in response to triggers, causes patients to experience similar symptoms to those of asthma. The gold standard for diagnosing ILO is direct visualisation of abnormal laryngeal movement by using laryngoscopy during an episode even though discerning the pathological laryngeal movements requires specialist training and is only available in specialist care. Questionnaires for diagnosing ILO are of limited utility though the Pittsburgh VCD Index has been proposed as a screening tool for ILO (62). *Research regarding the non-invasive tools for diagnosing ILO in primary care is needed*.

Once the traits of BPD and ILO have been diagnosed, they are treatable by specialist physiotherapists and speech therapists, respectively. The model of care for the treatment of BPD and ILO is usually through one-on-one sessions, with limited treatment capacity even in specialist care. Problematically, patients with BPD and ILO managed within specialist care are likely a small fraction of those within primary care—similar management could not feasibly be provided to the number of patients suspected within primary care. Video-based treatments for BPD have been investigated (63), and *more research on how a large number of patients with BPD and ILO might be treated in a resource-efficient manner in primary care is needed*.

### Management of complex breathlessness and SABA “addiction” in primary care

Whilst acute breathlessness during exacerbations is a characteristic feature of asthma exacerbations, chronic breathlessness is a feature of the symptom burden in a minority of asthmatic patients. Whilst this chronic breathlessness can be a feature of under-treated airway inflammation with on-going variable obstruction of the small airways, in many patients, chronic breathlessness is driven by other factors such as BPD, ILO, deconditioning, and obesity. Deconditioning—or in lay terms being “unfit” through lack of exercise over time—is common especially since the COVID-19 pandemic and can respond to advice, reassurance, and exercise therapy. Whilst identification and targeted treatment of each of these individual treatable traits is likely to be beneficial, resolution of the breathlessness is likely to require the combined treatment of all these treatable traits, i.e., through bundle interventions.

In the parallel condition of chronic obstructive pulmonary disease (COPD), the most-researched bundle intervention is pulmonary rehabilitation (PR), which combines a structured programme of self-management education and tailored exercise, delivered by a multidisciplinary team of practitioners. A growing evidence base for the benefits of PR in asthma exists (64, 65), but *further research regarding the components of PR required in asthma for optimal improvements in patient symptom burdens is needed*.

However, our experience is that in some patients reliance and overuse of SABA inhalers are not only disproportionate to other measures of asthma control but also to reported patient breathlessness. We would propose that some patients have gone on to develop a psychological dependency on their SABA inhalers and potentially addiction. *Research to understand and manage patients with psychological dependency and overuse of SABA inhalers despite controlled airway inflammation is now needed*.

### Management of extra-pulmonary treatable traits in primary care

Rhinitis is a common treatable trait in asthma and can be either allergic (seasonal or perennial) or non-allergic (66). Topical nasal steroid sprays can be beneficial in all patients with rhinitis, and, for those with allergic rhinitis, anti-histamines can be prescribed in primary care with advice on allergen avoidance. Whilst diagnosing nasal polyps requires imaging or nasoendoscopy, anosmia is a useful indicator of possible nasal polyp disease. Patients with severe asthma and nasal polyps often exhibit worsening of airway disease after exposure to salicylates including those in aspirin and non-steroidal anti-inflammatory drugs (NSAIDs). It is important that potential NSAID intolerance should be diagnosed early, and advice to avoid them should be suggested; otherwise, delays in such advice can lead to avoidable exacerbations (67).

Similar to poor adherence to preventer medications and rhinitis, smoking is associated with uncontrolled asthma (66). All healthcare workers should ask patients about their smoking habits and encourage those who smoke to stop including referral as appropriate to smoking cessation.

Primary care has extensive experience in managing corticosteroid-associated comorbidities such as obesity, gastro-oesophageal reflux, and osteoporosis. However, at referral to severe asthma centres, these comorbidities are highly prevalent and often untreated (47, 68), suggesting that they are often overlooked during primary care asthma reviews (rather than a lack of knowledge on how to treat within primary care).

Similarly, cumulative exposure to systemic corticosteroids in patients with uncontrolled asthma is associated with an increased prevalence of cardiovascular comorbidity (32), which may not be appreciated during routine asthma reviews. Anxiety and depression are also common in patients with uncontrolled asthma and other causes of asthma-like symptoms. This may be due to adverse effects of systemic corticosteroids, the impact of disabling breathlessness on patients’ quality of life, or direct interaction between disease pathophysiologies (32, 69). Importantly, these comorbidities can worsen the impact of asthma symptoms, and interventions for anxiety and depression have the potential to improve asthma symptom control (69). Our recent research shows the increased prevalence of anxiety and depression in non-eosinophilic asthmatic patients over-prescribed SABA inhalers (8).

Therefore, measures should be implemented in primary care to ensure these extra-pulmonary treatable traits are addressed. However, we need to be mindful of the pressures and workload primary care is under in many healthcare systems. Primary care practitioners are in many ways the experts in providing holistic care to patients with multiple comorbidities. We speculate that these extra-pulmonary comorbidities are often undiagnosed before referral to severe asthma centres due to being overlooked during asthma review rather than from primary care not being skilled to manage such conditions.

In primary care, long-term condition reviews often use structured templates, which can provide useful guidance on issues to be considered at review but can unintentionally restrict reviews to those topics included in templates (70). *Research is therefore needed into whether measures such as inclusion of extra-pulmonary treatable traits in templates for primary care asthma management are feasible and improve patient care*.

## Discussion

The adoption of the personalised, treatable traits approach has led to major health benefits for severe asthma patients managed at specialist centres and facilitated the “biologics era” with new, effective treatment options for patients previously deemed to have “treatment refractory” asthma. However, most asthma management in primary care tends to follow a “one-size-fits-all” approach emphasising a single pharmacotherapeutic ladder for patient management. This is associated with impaired care in patients whose symptoms and airway inflammation are discordant, and extra-pulmonary treatable traits can often be overlooked. We now believe that it is time that the treatable traits approach is embedded in primary care as many aspects could be rapidly implemented, even though much research is needed to better understand how assessment and treatment of some traits could be achieved at volume in primary care. All healthcare professionals caring for patients with asthma across care settings should become more aware of the treatable traits associated with asthma and the benefits gained from addressing all the traits. Ultimately, implementation of such a change in the paradigm of asthma care will take time and will need a shared journey with deeper collaboration between severe asthma centres and local primary care.

## Data Availability

The original contributions presented in the study are included in the article, further inquiries can be directed to the corresponding author.

## References

[1] Redmond C Heaney LG Chaudhuri R Jackson DJ Menzies-Gow A Pfeffer P Benefits of specialist severe asthma management: demographic and geographic disparities. Eur Respir J. (2022) 60(6):2200660. 10.1183/13993003.00660-2022 35777771 PMC9753476

[2] Denton E Lee J Tay T Radhakrishna N Hore-Lacy F Mackay A Systematic assessment for difficult and severe asthma improves outcomes and halves oral corticosteroid burden independent of monoclonal biologic use. J Allergy Clin Immunol Pract. (2020) 8(5):1616–24. 10.1016/j.jaip.2019.12.037 31954193

[3] Global Initiative for Asthma. Global strategy for asthma management and prevention (2023). Available at: https://ginasthma.org/wp-content/uploads/2023/07/GINA-2023-Full-report-23_07_06-WMS.pdf (Accessed July 27, 2023).

[4] British Thoracic Society, Health Improvement Scotland. SIGN 158: British guideline on the management of asthma (2019). Available at: https://www.brit-thoracic.org.uk/document-library/guidelines/asthma/btssign-guideline-for-the-management-of-asthma-2019 (Accessed July 27, 2023).

[5] Hekking PW Wener RR Amelink M Zwinderman AH Bouvy ML Bel EH. The prevalence of severe refractory asthma. J Allergy Clin Immunol. (2015) 135(4):896–902. 10.1016/j.jaci.2014.08.042 25441637

[6] Ryan D Heatley H Heaney LG Jackson DJ Pfeffer PE Busby J Potential severe asthma hidden in UK primary care. J Allergy Clin Immunol Pract. (2021) 9(4):1612–23. 10.1016/j.jaip.2020.11.053 33309935

[7] Pavord ID Beasley R Agusti A Anderson GP Bel E Brusselle G After asthma: redefining airways diseases. Lancet. (2018) 391(10118):350–400. 10.1016/S0140-6736(17)30879-6 28911920

[8] Pfeffer P Hajmohammadi H Cole J Griffiths C Hull S De Simoni A. Characteristics of patients with asthma overprescribed short-acting beta-agonist (SABA) reliever inhalers stratified by blood eosinophil count in north East London: a cross-sectional observational study. BJGP Open. (2023) 7(2):BJGPO.2023.0020. 10.3399/BJGPO.2023.0020 36921995 PMC10354394

[9] Toop L. Primary care: core values. Patient centred primary care. Br Med J. (1998) 316(7148):1882–3. 10.1136/bmj.316.7148.1882 9632410 PMC1113361

[10] Greenhalgh T Eversley J. Quality in general practice: towards a holistic approach. London: King’s Fund (1999).

[11] Royal College of General Practitioners, London. The power of relationships: what is relationship-based care and why is it important (2021)? Available at: https://www.rcgp.org.uk/getmedia/ca3e21e7-f742-47d7-9538-77e59bbb1ec7/power-of-relationships-rcgp-2021.pdf (Accessed July 27, 2023).

[12] Kerkhof M Tran TN Allehebi R Canonica GW Heaney LG Hew M Asthma phenotyping in primary care: applying the international severe asthma registry eosinophil phenotype algorithm across all asthma severities. J Allergy Clin Immunol Pract. (2021) 9(12):4353–70. 10.1016/j.jaip.2021.07.056 34403837

[13] Heaney LG Perez de Llano L Al-Ahmad M Backer V Busby J Canonica GW Eosinophilic and noneosinophilic asthma: an expert consensus framework to characterize phenotypes in a global real-life severe asthma cohort. Chest. (2021) 160(3):814–30. 10.1016/j.chest.2021.04.013 33887242

[14] Flood-Page P Swenson C Faiferman I Matthews J Williams M Brannick L A study to evaluate safety and efficacy of mepolizumab in patients with moderate persistent asthma. Am J Respir Crit Care Med. (2007) 176(11):1062–71. 10.1164/rccm.200701-085OC 17872493

[15] Haldar P Brightling CE Hargadon B Gupta S Monteiro W Sousa A Mepolizumab and exacerbations of refractory eosinophilic asthma. N Engl J Med. (2009) 360(10):973–84. 10.1056/NEJMoa0808991 19264686 PMC3992367

[16] Pavord ID Korn S Howarth P Bleecker ER Buhl R Keene ON Mepolizumab for severe eosinophilic asthma (DREAM): a multicentre, double-blind, placebo-controlled trial. Lancet. (2012) 380(9842):651–9. 10.1016/S0140-6736(12)60988-X 22901886

[17] Price DB Rigazio A Campbell JD Bleecker ER Corrigan CJ Thomas M Blood eosinophil count and prospective annual asthma disease burden: a UK cohort study. Lancet Respir Med. (2015) 3(11):849–58. 10.1016/S2213-2600(15)00367-7 26493938

[18] Pavord ID Deniz Y Corren J Casale TB FitzGerald JM Izuhara K Baseline FeNO independently predicts the dupilumab response in patients with moderate-to-severe asthma. J Allergy Clin Immunol Pract. (2023) 11(4):1213–20. 10.1016/j.jaip.2022.11.043 36535524

[19] Butler CA Heaney LG. Fractional exhaled nitric oxide and asthma treatment adherence. Curr Opin Allergy Clin Immunol. (2021) 21(1):59–64. 10.1097/ACI.0000000000000704 33369570

[20] Rupani H Kent BD. Using fractional exhaled nitric oxide measurement in clinical asthma management. Chest. (2022) 161(4):906–17. 10.1016/j.chest.2021.10.015 34673021

[21] Kamat S Gouia I Chao J Small M Khan A Siddall J. Availability of fractional exhaled nitric oxide (FeNO) and eosinophil (EOS) count data among patients with severe asthma in five European countries. J Allergy Clin Immunol Pract. (2020) 145(2 Suppl): AB205. 10.1016/j.jaci.2019.12.214

[22] The AHSN Network. Improving access to FeNO testing in primary care (2023). Available at: https://www.ahsnnetwork.com/programmes/respiratory-disease/bettering-access-to-feno-testing-in-primary-care/#:∼:text=An%20estimated%2053%25%20of%20Primary,asthmatics%20faster%20and%20more%20accurately (Accessed July 27, 2023).

[23] Lo D Beardsmore C Roland D Richardson M Yang Y Danvers L Spirometry and FeNO testing for asthma in children in UK primary care: a prospective observational cohort study of feasibility and acceptability. Br J Gen Pract. (2020) 70(700):e809–16. 10.3399/bjgp20X713033 33077507 PMC7575406

[24] Clark VL Gibson PG Genn G Hiles SA Pavord ID McDonald VM. Multidimensional assessment of severe asthma: a systematic review and meta-analysis. Respirology. (2017) 22(7):1262–75. 10.1111/resp.13134 28776330

[25] McDonald VM Harrington J Clark VL Gibson PG. Multidisciplinary care in chronic airway diseases: the Newcastle model. ERJ Open Res. (2022) 8(3):00215–2022. 10.1183/23120541.00215-2022 35983538 PMC9379354

[26] Agusti A Bel E Thomas M Vogelmeier C Brusselle G Holgate S Treatable traits: toward precision medicine of chronic airway diseases. Eur Respir J. (2016) 47(2):410–9. 10.1183/13993003.01359-2015 26828055

[27] Byrne C Pfeffer PE De Simoni A. Experiences of diagnosis, symptoms and use of reliever inhalers in patients with asthma and concurrent inducible laryngeal obstruction (ILO) or breathing pattern disorder (BPD): qualitative analysis of a UK asthma online community. J Med Internet Res. (2023) 25:e44453. 10.2196/44453 37578820 PMC10463086

[28] Crawford AL Blakey JD Baumwol K. Paroxysmal dyspnoea in asthma: wheeze, ILO or dysfunctional breathing? Front Allergy. (2022) 3:1054791. 10.3389/falgy.2022.1054791 36465884 PMC9712793

[29] Robinson DS Campbell DA Durham SR Pfeffer J Barnes PJ Chung KF. Systematic assessment of difficult-to-treat asthma. Eur Respir J. (2003) 22(3):478–83. 10.1183/09031936.03.00017003 14516138

[30] Haldar P Pavord ID Shaw DE Berry MA Thomas M Brightling CE Cluster analysis and clinical asthma phenotypes. Am J Respir Crit Care Med. (2008) 178(3):218–24. 10.1164/rccm.200711-1754OC 18480428 PMC3992366

[31] Waljee AK Rogers MA Lin P Singal AG Stein JD Marks RM Short term use of oral corticosteroids and related harms among adults in the United States: population based cohort study. Br Med J. (2017) 357:j1415. 10.1136/bmj.j1415 28404617 PMC6284230

[32] Price DB Trudo F Voorham J Xu X Kerkhof M Ling Zhi Jie J Adverse outcomes from initiation of systemic corticosteroids for asthma: long-term observational study. J Asthma Allergy. (2018) 11:193–204. 10.2147/JAA.S176026 30214247 PMC6121746

[33] Green RH Brightling CE McKenna S Hargadon B Parker D Bradding P Asthma exacerbations and sputum eosinophil counts: a randomised controlled trial. Lancet. (2002) 360(9347):1715–21. 10.1016/S0140-6736(02)11679-5 12480423

[34] Wagener AH de Nijs SB Lutter R Sousa AR Weersink EJ Bel EH External validation of blood eosinophils, FE(NO) and serum periostin as surrogates for sputum eosinophils in asthma. Thorax. (2015) 70(2):115–20. 10.1136/thoraxjnl-2014-205634 25422384

[35] Heaney LG Busby J Hanratty CE Djukanovic R Woodcock A Walker SM Composite type-2 biomarker strategy versus a symptom-risk-based algorithm to adjust corticosteroid dose in patients with severe asthma: a multicentre, single-blind, parallel group, randomised controlled trial. Lancet Respir Med. (2021) 9(1):57–68. 10.1016/S2213-2600(20)30397-0 32916135 PMC7783382

[36] Kachroo P Stewart ID Kelly RS Stav M Mendez K Dahlin A Metabolomic profiling reveals extensive adrenal suppression due to inhaled corticosteroid therapy in asthma. Nat Med. (2022) 28(4):814–22. 10.1038/s41591-022-01714-5 35314841 PMC9350737

[37] Andrejak C Nielsen R Thomsen V Duhaut P Sorensen HT Thomsen RW. Chronic respiratory disease, inhaled corticosteroids and risk of non-tuberculous mycobacteriosis. Thorax. (2013) 68:256–62. 10.1136/thoraxjnl-2012-201772 22781123

[38] Barnes PJ. Corticosteroid resistance in patients with asthma and chronic obstructive pulmonary disease. J Allergy Clin Immunol. (2013) 131(3):636–45. 10.1016/j.jaci.2012.12.1564 23360759

[39] Lu J Xiong L Zhang X Liu Z Wang S Zhang C The role of lower airway dysbiosis in asthma: dysbiosis and asthma. Mediators Inflamm. (2017) 2017:3890601. 10.1155/2017/3890601 29386750 PMC5745728

[40] Hull JH. Not all wheeze is asthma: time for patients to exercise their rights. Thorax. (2015) 70(1):7–8. 10.1136/thoraxjnl-2014-206096 25398497

[41] Gibson PG McDonald VM Granchelli A Olin JT. Asthma and comorbid conditions-pulmonary comorbidity. J Allergy Clin Immunol Pract. (2021) 9(11):3868–75. 10.1016/j.jaip.2021.08.028 34492401

[42] De Simoni A Hajmohammadi H Pfeffer P Cole J Griffiths C Hull SA. Reducing short-acting beta-agonist overprescribing in asthma: lessons from a quality-improvement prescribing project in east London. Br J Gen Pract. (2022) 72(722):e619–26. 10.3399/BJGP.2021.0725 35995577 PMC9423045

[43] Taylor DR. The beta-agonist saga and its clinical relevance: on and on it goes. Am J Respir Crit Care Med. (2009) 179(11):976–8. 10.1164/rccm.200901-0055CC 19286624

[44] Lai CC Chen CH Wang YH Wang CY Wang HC. The impact of the overuse of short-acting β2-agonists on the risk of sepsis and septic shock. J Allergy Clin Immunol. (2022) 150(1):75–81. 10.1016/j.jaci.2021.11.029 35108605

[45] Khan MA Howell A Pham T Guzman N. Reverse Takotsubo cardiomyopathy in the setting of acute asthma exacerbation. Cureus. (2021) 13(6):e15469. 10.7759/cureus.15469 34262807 PMC8260191

[46] Ahmadi Z. The burden of chronic breathlessness across the population. Curr Opin Support Palliat Care. (2018) 12(3):214–8. 10.1097/SPC.0000000000000364 29927754

[47] Jackson DJ Busby J Pfeffer PE Menzies-Gow A Brown T Gore R Characterisation of patients with severe asthma in the UK severe asthma registry in the biologic era. Thorax. (2021) 76(3):220–7. 10.1136/thoraxjnl-2020-215168 33298582 PMC7892381

[48] Chalitsios CV McKeever TM Shaw DE. Incidence of osteoporosis and fragility fractures in asthma: a UK population-based matched cohort study. Eur Respir J. (2021) 57(1):2001251. 10.1183/13993003.01251-2020 32764111

[49] Schlaich C Minne HW Bruckner T Wagner G Gebest HJ Grunze M Reduced pulmonary function in patients with spinal osteoporotic fractures. Osteoporos Int. (1998) 8(3):261–7. 10.1007/s001980050063 9797911

[50] Dixon AE Peters U. The effect of obesity on lung function. Expert Rev Respir Med. (2018) 12(9):755–67. 10.1080/17476348.2018.1506331 30056777 PMC6311385

[51] Scadding G Walker S. Poor asthma control?—then look up the nose. The importance of co-morbid rhinitis in patients with asthma. Prim Care Respir J. (2012) 21(2):222–8. 10.4104/pcrj.2012.00035 22643359 PMC6547933

[52] Lee LA Bailes Z Barnes N Boulet LP Edwards D Fowler A Efficacy and safety of once-daily single-inhaler triple therapy (FF/UMEC/VI) versus FF/VI in patients with inadequately controlled asthma (CAPTAIN): a double-blind, randomised, phase 3A trial. Lancet Respir Med. (2021) 9(1):69–84. 10.1016/S2213-2600(20)30389-1 32918892

[53] Honkoop PJ Loijmans RJ Termeer EH Snoeck-Stroband JB van den Hout WB Bakker MJ Symptom- and fraction of exhaled nitric oxide-driven strategies for asthma control: a cluster-randomized trial in primary care. J Allergy Clin Immunol. (2015) 135(3):682–8. 10.1016/j.jaci.2014.07.016 25174865

[54] Papi A Virchow JC Singh D Kots M Vele A Georges G Extrafine triple therapy and asthma exacerbation seasonality: TRIMARAN and TRIGGER post hoc analyses. J Allergy Clin Immunol. (2021) 148(1):262–5. 10.1016/j.jaci.2021.01.007 33485959

[55] Shim JS Jin J Kim SH Lee T Jang AS Park CS Clinical predictors of treatment response to tiotropium add-on therapy in adult asthmatic patients: from multicenter real-world cohort data in Korea. World Allergy Organ J. (2022) 15(12):100720. 10.1016/j.waojou.2022.100720 36438190 PMC9679363

[56] Mabotuwana T Warren J Harrison J Kenealy T. What can primary care prescribing data tell us about individual adherence to long-term medication?—comparison to pharmacy dispensing data. Pharmacoepidemiol Drug Saf. (2009) 18(10):956–64. 10.1002/pds.1803 19609958

[57] Boulding R Stacey R Niven R Fowler SJ. Dysfunctional breathing: a review of the literature and proposal for classification. Eur Respir Rev. (2016) 25(141):287–94. 10.1183/16000617.0088-2015 27581828 PMC9487208

[58] Courtney R van Dixhoorn J Cohen M. Evaluation of breathing pattern: comparison of a manual assessment of respiratory motion (MARM) and respiratory induction plethysmography. Appl Psychophysiol Biofeedback. (2008) 33(2):91–100. 10.1007/s10484-008-9052-3 18320303

[59] Ionescu MF Mani-Babu S Degani-Costa LH Johnson M Paramasivan C Sylvester K Cardiopulmonary exercise testing in the assessment of dysfunctional breathing. Front Physiol. (2020) 11:620955. 10.3389/fphys.2020.620955 33584339 PMC7873943

[60] Todd S Walsted ES Grillo L Livingston R Menzies-Gow A Hull JH. Novel assessment tool to detect breathing pattern disorder in patients with refractory asthma. Respirology. (2018) 23(3):284–90. 10.1111/resp.13173 28905471

[61] Bondarenko J Hew M Button B Webb E Jackson V Clark R Reliability of the breathing pattern assessment tool for in-person or remote assessment in people with asthma. Clin Exp Allergy. (2021) 51(9):1218–20. 10.1111/cea.13856 33638261

[62] Traister RS Fajt ML Landsittel D Petrov AA. A novel scoring system to distinguish vocal cord dysfunction from asthma. J Allergy Clin Immunol Pract. (2014) 2(1):65–9. 10.1016/j.jaip.2013.09.002 24565771

[63] Thomas M Bruton A Little P Holgate S Lee A Yardley L A randomised controlled study of the effectiveness of breathing retraining exercises taught by a physiotherapist either by instructional DVD or in face-to-face sessions in the management of asthma in adults. Health Technol Assess. (2017) 21(53):1–162. 10.3310/hta21530 PMC5632761 28944752

[64] Zampogna E Spanevello A Visca D. Pulmonary rehabilitation: promising nonpharmacological approach for treating asthma? Curr Opin Allergy Clin Immunol. (2020) 20(1):80–4. 10.1097/ACI.0000000000000597 31633568

[65] Feng Z Wang J Xie Y Li J. Effects of exercise-based pulmonary rehabilitation on adults with asthma: a systematic review and meta-analysis. Respir Res. (2021) 22(1):33. 10.1186/s12931-021-01627-w 33516207 PMC7847170

[66] Clatworthy J Price D Ryan D Haughney J Horne R. The value of self-report assessment of adherence, rhinitis and smoking in relation to asthma control. Prim Care Respir J. (2009) 18(4):300–5. 10.4104/pcrj.2009.00037 19562233 PMC6619365

[67] Kshirsagar RS Chou DW Wei J Liang J. Aspirin-exacerbated respiratory disease: longitudinal assessment of a large cohort and implications of diagnostic delay. Int Forum Allergy Rhinol. (2020) 10(4):465–73. 10.1002/alr.22516 32104978

[68] Janssen SMJ van Helvoort HAC Tjalma TA Antons JC Djamin RS Simons SO Impact of treatable traits on asthma control and quality of life. J Allergy Clin Immunol Pract. (2023) 11(6):1823–33. 10.1016/j.jaip.2023.02.034 36893847

[69] Cooley C Park Y Ajilore O Leow A Nyenhuis SM. Impact of interventions targeting anxiety and depression in adults with asthma. J Asthma. (2022) 59(2):273–87. 10.1080/02770903.2020.1847927 33176512 PMC8221364

[70] Morrissey M Shepherd E Kinley E McClatchey K Pinnock H. Effectiveness and perceptions of using templates in long-term condition reviews: a systematic synthesis of quantitative and qualitative studies. Br J Gen Pract. (2021) 71(710):e652–e9. 10.3399/BJGP.2020.0963 33690148 PMC8321439

